# Implementation of post-discharge malaria chemoprevention (PDMC) in Benin, Kenya, Malawi, and Uganda: stakeholder engagement meeting report

**DOI:** 10.1186/s12936-023-04810-0

**Published:** 2024-03-27

**Authors:** Jenny Hill, PDMC Saves Lives Consortium, Manfred Accrombessi, Valérie Briand, Aggrey Dhabangi, Jenny Hill, Jenna Hoyt, Richard Idro, Carole Khairallah, Simon Kariuki, Feiko O. ter Kuile, Titus Kwambai, Adrian J. F. Luty, Lucinda Manda-Taylor, Achille Massougbodji, Juliet Otieno, Kamija S. Phiri, Laura Rosu, Joseph Rujumba, Tracy Seddon, Brian Tangara, Jeanne Perpétue Vincent, Eve Worrall

**Affiliations:** 1 Institut de Recherche Clinique du Benin (IRCB), Cotonou, Benin; 2 Kenya Medical Research Institute (KEMRI), Kisumu, Kenya; 3 KEMRI/Wellcome Trust, Kilifi, Kenya; 4 Makerere University College of Health Sciences, Kampala, Uganda; 5 Training and Research Unit of Excellence (TRUE), Blantyre, Malawi; 6 Kamuzu University of Health Sciences, Blantyre, Malawi; 7 Centers for Disease Control and Prevention (CDC), Atlanta, US; 8 MERIT, IRD, Université Paris Cité, Paris, France; 9 Epicentre, Paris, France; 10 Department of Clinical Sciences, Liverpool School of Tropical Medicine, Liverpool, L3 5QA UK

**Keywords:** Post-discharge malaria chemoprevention (PDMC), Dihydroartemisinin-piperaquine (DP), Artemether-lumefantrine (AL), Sulfadoxine-pyrimethamine (SP), Sulfadoxine-pyrimethamine and amodiaquine (SP-AQ), Artesunate-amodiaquine (AS-AQ), Implementation research, Formative research

## Abstract

A Stakeholder engagement meeting on the implementation of post-discharge malaria chemoprevention (PDMC) in Benin, Kenya, Malawi, and Uganda was held in Nairobi, Kenya, on 27 September 2023. Representatives from the respective National Malaria Control Programmes, the World Health Organization (WHO) Geneva, Africa Regional and Kenya offices, research partners, non-governmental organizations, and the Medicines for Malaria Venture participated. PDMC was recommended by the WHO in June 2022 and involves provision of a full anti-malarial treatment course at regular intervals during the post-discharge period in children hospitalized with severe anaemia in areas of moderate-to-high malaria transmission. The WHO recommendation followed evidence from a meta-analysis of three clinical trials and from acceptability, delivery, cost-effectiveness, and modelling studies. The trials were conducted in The Gambia using monthly sulfadoxine-pyrimethamine during the transmission season, in Malawi using monthly artemether-lumefantrine, and in Kenya and Uganda using monthly dihydroartemisinin-piperaquine, showing a significant reduction in all-cause mortality by 77% (95% CI 30–98) and a 55% (95% CI 44–64) reduction in all-cause hospital readmissions 6 months post-discharge. The recommendation has not yet been implemented in sub-Saharan Africa. There is no established platform for PDMC delivery. The objectives of the meeting were for the participating countries to share country contexts, plans and experiences regarding the adoption and implementation of PDMC and to explore potential delivery platforms in each setting. The meeting served as the beginning of stakeholder engagement within the PDMC Saves Lives project and will be followed by formative and implementation research to evaluate alternative delivery strategies in selected countries. Meeting highlights included country consensus on use of dihydroartemisinin-piperaquine for PDMC and expansion of the target group to "severe anaemia *or severe malaria*", in addition to identifying country-specific options for PDMC delivery for evaluation in implementation research. Further exploration is needed on whether the age group should be extended to school-age children.

## Background

On 3 June 2022, the World Health Organization (WHO) updated the malaria chemoprevention guidelines to include post-discharge malaria chemoprevention (PDMC) as a new intervention for the post-discharge management of hospitalized children with severe anaemia in settings with moderate to high malaria transmission. The PDMC strategy aims to reduce readmission and death post-discharge by administering full treatment courses of long-acting anti-malarials at pre-defined time intervals to children under five recently discharged from hospital after recovery from severe anaemia, irrespective of a patient’s malaria status. The recommendation for PDMC [1] was based on a meta-analysis of three double-blind placebo-controlled PDMC trials involving 3663 children with severe anaemia which showed that 3 months of PDMC was associated with a 77% (95% CI 30–98) reduction in mortality during the intervention period (primary outcome) (p = 0.009) and a 55% (95% CI 44–64) reduction in all-cause readmissions (p < 0.001) 6 months post-discharge [2]. The trials used three alternative drug regimens—monthly sulfadoxine-pyrimethamine (SP) until the end of the malaria transmission season (average: 3.1 doses per child) (N = 1200, the Gambia [3]), monthly artemether-lumefantrine (AL) given at 4 and 8 weeks post-discharge (N = 1414, Malawi [4]), or monthly dihydroartemisinin-piperaquine (DP) given at the end of the 2nd, 6th, and 10th-week post-discharge (N = 1049, Uganda and Kenya [5]). Evidence from additional studies on acceptability [6], delivery strategies [7], cost-effectiveness [8], and modelling [9] were also reviewed. The WHO recommendation stopped short of recommending which anti-malarial drug should be used and how best to deliver PDMC, indicating that these decisions should be made at the national level and adapted to suit local contexts. In addition, the recommendation highlighted the need for implementation research on optimal delivery strategies for PDMC to help guide decision-making.

This stakeholder engagement meeting was convened by the PDMC Saves Lives project, funded by the Global Health European and Developing Countries Clinical Trials Partnership (EDCTP-3) Joint Undertaking and its members. The project aims to generate evidence on the optimal delivery platform(s) for PDMC and accelerate policy adoption by national control programmes in malaria-endemic countries. Evidence will be generated through formative research in Benin, Kenya, Malawi, and Uganda, followed by implementation trials in Benin and Kenya. The trials will be co-designed with a range of stakeholders, including caregivers, and will evaluate the feasibility and acceptability of at least two alternative delivery strategies and determine cost-effectiveness.

## Meeting objectives

1. To review and discuss the evidence for PDMC with country stakeholders.
2. To learn about country contexts, plans and experiences regarding the adoption and implementation of PDMC.
3. To identify health system constraints specific to each country and explore potential solutions for scaling up PDMC in the region.
4. To develop consensus on indicators for Health Management Information Systems (HMIS).
5. To identify potential delivery strategies for the evaluation of PDMC.

Participants included representatives from the respective National Malaria Control Programmes (NMCPs) in Benin, Kenya, Malawi and Uganda, the World Health Organization offices in Geneva, the Africa Regional Office (AFRO) and Kenya, research partners from the PDMC Saves Lives project (Training and Research Unit of Excellence (TRUE), Blantyre, Malawi); Makerere University, Kampala, Uganda; Kenya Medical Research Institute (KEMRI), Kisumu, Kenya; U.S. Centers for Disease Control and Prevention (CDC), Atlanta, US; Institut de Recherche Clinique du Bénin (IRCB), Cotonou, Benin; African Medical and Research Foundation (AMREF) Health Africa, Nairobi, Kenya; Malaria Consortium, Kampala, Uganda; Centre Hospitalier de la Mère et de l’enfant (CHU-MEL), Cotonou, Benin; President’s Malaria Initiative (PMI), Nairobi, Kenya; the Medicines for Malaria Venture (MMV), Geneva, Switzerland; Epicentre, Paris, France; Institut de recherche pour le development (IRD), Paris, France; and the Liverpool School of Tropical Medicine (LSTM), UK.

## Meeting proceedings

The one-day meeting was conducted at the Fairview Hotel in Nairobi, Kenya, with several participants joining the meeting online. All sessions were conducted in English and French with simultaneous translation. The morning sessions focussed on a review of the evidence for PDMC, a review of the WHO recommendation on PDMC and a review of the country contexts, plans and experiences regarding the adoption and implementation of PDMC, chaired by Dr Joseph Rujumba from Makerere University, Uganda. The afternoon sessions consisted of breakout groups to explore potential delivery platforms, considering the different health systems and epidemiological settings in each country, and priority areas for formative and implementation research, chaired by Professor Kamija Phiri, Director of the Training and Research Unit of Excellence (TRUE), Malawi. The meeting served as the beginning of longer-term stakeholder engagement within the PDMC Saves Lives project and will be followed by a formative research phase and implementation trials to evaluate alternative delivery strategies in selected countries.

The meeting participants were welcomed by Dr Kibor Keitany, Head of the National Malaria Control Programme in Kenya, followed by opening remarks provided virtually by the Principal Secretary of the State Department of Public Health and Professional Standards, Mary Muthoni, HSC. In her remarks, she emphasized the importance of investment, innovation, and implementation—including augmenting existing interventions and supporting research into more impactful interventions. She also noted that important research findings, such as those for PDMC, should be swiftly incorporated into policies and strategies to improve the quality of health care for Kenyans.

The meeting outcomes and implementation research questions are summarized in the conclusion section of this report.

## Session 1. Review of the evidence on PMDC

### Summary of evidence on PDMC—Speaker: Prof Kamija Phiri, TRUE, Malawi

The evidence for PDMC began with studies in Malawi, where severe anaemia accounts for 30% of hospital admissions and 6% of in-hospital mortality. Post-discharge mortality by 6 months was found to be five times higher among children admitted with anaemia than hospital controls without severe anaemia (1.6%) and the risk of rehospitalization within 6 months was 93%, predominantly due to all-cause severe anaemia [10]. Severe anaemia has a complex aetiology and malaria is an important factor. A proof of principal trial in Malawi of intermittent preventive therapy with monthly AL for the post-discharge management of severe anaemia in children aged 4–59 months with confirmed malaria showed a 41% reduction in deaths or readmission due to severe anaemia/severe malaria, a 38% reduction in all-cause hospitalization and a 49% reduction in clinical malaria, however, protection began to wane after 3 months [4]. This was followed by confirmatory trials in Kenya and Uganda using a longer-acting artemisinin-based combination therapy (ACT), DP, in children under 5 years old hospitalized with severe anaemia. This trial showed a 92% reduction in all-cause mortality and 69% reduction in all-cause rehospitalization during the intervention period [5]. A subsequent individual-participant data meta-analysis of PDMC trials included an older trial with monthly SP provided during the malaria transmission season in The Gambia [3], Malawi with AL [4], and Kenya/Uganda with DP [5] and showed a significant 77% reduction in all-cause mortality and a 55% reduction in all cause readmissions [2]. These findings were highly malaria-specific and the economic analysis suggests PDMC is likely to be a cost-saving intervention [8]. Evidence from a randomized control trial in Malawi on the feasibility of different delivery strategies indicated that the provision of all PDMC drugs to the caregiver on discharge was associated with improved adherence (24%) when compared to facility-based delivery, which required caregivers to return to the facility for monthly PDMC courses [7]. This finding was reinforced by an acceptability study where caregivers expressed a preference for receiving all medicines on discharge, and, interestingly, indicated that short message services (SMS) reminders were not needed as they felt the dates given in the maternal and child health booklet were sufficient [6].

### WHO Malaria Guideline on Post discharge Malaria Chemoprevention—Speaker: Dr Peter Olumese, WHO Global Malaria Programme, Geneva

In 2022, WHO updated the chemoprevention guidelines to include the provision of a full treatment course of antimalarials at regular intervals as part of the post-discharge management for children in areas of moderate to high transmission hospitalized with severe anaemia [1]. The aim of PDMC is to prevent new malaria infections during the high-risk post-discharge period and thereby reduce re-admissions and death. Given the complexity of severe anaemia aetiology, PDMC is to be provided irrespective of the aetiology except when it is due to blood loss following trauma, surgery, malignancy, sickle cell anaemia or a bleeding disorder. The recommendation suggests AL, DP and SP are effective for use as PDMC, but cautions against using drugs that are currently used as first line treatment for uncomplicated malaria. At present, guidance on how to implement PDMC is limited, and national malaria programmes are encouraged to use local data and tailor delivery strategies to reflect the country context. However, the WHO plans to publish an implementation manual in due course. Considerable research gaps related to PDMC have been identified. Namely, the optimal duration of PDMC across different transmission settings, patient adherence to PDMC, feasibility and cost-effectiveness of different delivery strategies and the feasibility of co-implementing PDMC with other chemoprevention interventions e.g., seasonal malaria chemoprevention (SMC), perennial malaria chemoprevention (PMC).

## Session 2. Country contexts, plans and experiences regarding PDMC adoption and implementation

A central aim of the stakeholder engagement meeting was to bring together countries that are at various stages of PDMC planning, implementation, and policy adoption so that experiences and learnings can be shared by those countries further along the pathway. Representatives from Ministries of Health (MoH) in Uganda, Malawi, Kenya, and Benin delivered presentations highlighting key contextual factors that relate to PDMC and provided a status update on the country’s PDMC policy adoption process and planning (Table 1).

**Table 1:** Country malaria treatment policy contexts and status of PDMC policy adoption

**Uganda**	
Treatment policy	1st line treatment for uncomplicated malaria Artemether-lumefantrine (AL) Alternative 1st line treatment is Artesunate-amodiaquine (AS-AQ) 2nd line treatment for uncomplicated malaria Dihydroartemisinin-piperaquine (DP) Severe malaria a) At health facility level: IV artesunate Dihydroartemisinin-piperaquine to be administered at discharge b) Pre-referral treatment at community and lower-level health facilities Rectal artesunate, then continue as in (a) above
PDMC policy adoption status	MoH has recommended adoption of WHO guidelines on PDMC
**Malawi**	
Treatment policy	1st line treatment for uncomplicated malaria Artemether-lumefantrine (AL) Dihydroartemisinin-piperaquine (DP) is set to replace AL as first line treatment but has not yet been rolled-out 2nd line treatment for uncomplicated malaria Artesunate-amodiaquine (AS-AQ) Severe malaria IV artesunate Dihydroartemisinin-piperaquine to be administered at discharge
PDMC policy adoption status	Evidence has been presented to Technical Working Group (TWG) (including case management) and Malaria Advisory Committee MoH has asked Health Service Delivery TWG to review results prior to giving policy approval
**Kenya**	
Treatment policy	1st line treatment for uncomplicated malaria Artemether-lumefantrine (AL) No planned changes to first line treatment choice 2nd line treatment for uncomplicated malaria Dihydroartemisinin-piperaquine (DP) Severe malaria IV artesunate Artemether-lumefantrine to be administered at discharge
PDMC policy adoption status	Policy adoption of PDMC has not yet been initiated Stakeholder engagement has commenced
**Benin**	
Treatment policy	1st line treatment for uncomplicated malaria Artemether-lumefantrine (AL) Dihydroartemisinin-piperaquine (DP) is used in private facilities but is not available in the public system 2nd line treatment for uncomplicated malaria Pyronaridine-artesunate (AP) but not yet in the supply chain Severe malaria IV artesunate Artemether-lumefantrine to be administered at discharge
PDMC policy adoption status	Policy adoption of PDMC has not yet been initiated Stakeholder engagement has commenced

### Uganda (Speaker: Dr Anthony Nuwa, Malaria Consortium)

In Uganda, the MoH has adopted all the WHO recommended chemoprevention interventions including the PDMC, making it the furthest along the policy pathway. PDMC with DP will be provided alongside a comprehensive package called *Smart Discharge* that will include health education on malaria prevention, provision of a long-lasting insecticide-treated net, and community follow-up by the Village Health Teams (VHTs). Plans are underway to integrate PDMC into the malaria case management guidelines and disseminate them nationally through circulars, training, and mentorships. Uganda has opted for a facility-based delivery strategy; healthcare provider training, provision of job aids and community sensitizations with VHTs will be carried out prior to implementation. In addition, DP for PDMC will be included in the general malaria commodity quantification.

### Malawi (Speaker: Dr Lumbani Munthali, NMCP)

In Malawi, a recent co-design workshop brought together key stakeholders to establish drug choice and optimal delivery strategies for PDMC. Importantly, given the country’s recent shift from AL to DP as first-line treatment for uncomplicated malaria, Malawi has opted for DP, having identified drug continuity post-discharge as key to optimizing implementation. The policy has been endorsed by the technical working group (TWG) for malaria case management and the National Malaria Advisory Committee Malawi further opted to use the term Post discharge malaria continuum of care (PDMCC) as this intervention was perceived as a continuation of management of the initial severe anaemia event that brought the child to hospital.

### Kenya (Speaker: Regina Kandie, NMCP)

Policy adoption in Kenya has yet to be initiated, however, the malaria control programme has expressed interest in exploring how PDMC could best be delivered. Co-design workshops with a range of key stakeholder groups will be conducted to select at least two delivery strategies that will subsequently be tested in the upcoming implementation trial. The outcome of this trial is anticipated to catalyse policy adoption in Kenya.

### Benin (Speaker: Dr Manfred Accrombessi, IRCB, on behalf of Dr Cyriaque Affoukou, NMCP)

The policy adoption process in Benin has not yet begun, however, the national malaria programme is interested in evaluating what delivery strategies would best suit the Benin context. Co-design workshops with stakeholders will be undertaken to select at least two delivery strategies to test through the forthcoming implementation trial. The outcome of this trial is anticipated to catalyse policy adoption in Benin.

## Session 3. Health system and contextual considerations specific to each country and potential delivery platforms for PDMC

During this session, participants formed ‘breakout groups’ by country, including MoH and research partners, for a facilitated discussion to consider their drug of choice for PDMC and potential delivery strategies. Groups were provided with a breakout session guide to consider specific questions captured in Appendix 1.

### Choice of PDMC drug

The WHO guidelines indicate that the PDMC trials were conducted with AL, DP, or SP. Of the three, DP showed the greatest efficacy in reducing all-cause mortality, whereas AL has the shortest duration of post-treatment prophylaxis [2]. However, WHO does not restrict the choice of antimalarials to these three drugs. SP combined with amodiaquine (SPAQ), commonly used for SMC, is another combination that could be considered for PDMC, especially in West Africa which has low levels of parasite resistance to SP. Participants discussed a range of views around drug choice for PDMC that focused on: drug procurement, supply chain and cost, concerns about drug resistance and, importantly, how to resolve the tension between adhering to the WHO guidance and considerations that will facilitate operationalizing PDMC in the context of a continuum of care.

### Key considerations discussed

1. Strong preference from participants for use of DP for PDMC in East and Southern Africa (both Malawi and Uganda have selected DP already as the drug of choice), main reasons being:
  - Efficacious, long half-life (protective cover for 1 month).
  - Ease of administration (1 × per day dosing).
  - Ease of implementation (already in use, known to healthcare providers).
  - Not currently used as first line treatment or for SMC (Uganda).
  - To be used as part of a continuum of care (in Malawi, Table 2).

**Table 2:** Malawi’s experience in decision-making for PDMC drug choice

Experience from Malawi: Post discharge malaria continuum of care	
In May 2023, stakeholders in Malawi (including the Ministry of Health) held a co-design workshop to determine the preferred delivery strategy and ACT for PDMC. A key output of that workshop was the decision to re-frame PDMC as ‘post-discharge malaria continuum of care (PDMCC)’ to reconcile the tension between WHO guidance on PDMC and implementation feasibility. Under this strategy, Malawi has opted to use DP for PDMC, which is also going to be the first-line treatment for uncomplicated malaria and the drug with which children will be discharged from hospital. The rationale was that it would ensure a continuum of care—with caregiver and healthcare providers already familiar with DP dosing. In addition, using DP post-discharge would likely create fewer procurement and supply chain complications
2. Drug preference for DP may conflict with the WHO recommendation to avoid selecting antimalarial drugs that are currently in use for first line malaria treatment; also need to consider drugs currently in use for other chemoprevention interventions (e.g., SMC, PMC, intermittent preventive treatment in pregnancy (IPTp)).
3. Preference for using a drug that promotes continuity of care—so the drug used at discharge is the same as the PDMC drug choice.
4. Drug choice has implications for procurement, supply chain management, and healthcare provider decision-making, points raised included:
  - Healthcare providers prioritizing drugs for different interventions (e.g., DP for treatment over PDMC).
  - Artificial stock outs created by healthcare providers opting to ‘hold back’ drugs for specific uses when stocks are running low (e.g., not using DP for PDMC to ensure availability for 1st line treatment).
5. PDMC adding pressure to key antimalarial drugs, contributing to resistance.
  - Points were made by several participants that the actual numbers of children requiring PDMC would be relatively low in each country (3000 in Malawi representing 0.03% of all malaria treatments) and as such use for PDMC is unlikely to be a driver of drug resistance.
6. Cross-border considerations: communities that access health services across two different countries might be given different drugs.
7. Other candidates for alternative PDMC drugs: Artesunate-amodiaquine (ASAQ), sulfadoxine-pyrimethamine with amodiaquine (SPAQ).
  - Drugs not currently used for first line treatment of uncomplicated malaria (i.e., does not contradict WHO recommendations).
  - Drugs already in use in-country—either for SMC or second line treatment options—and as such less obstacles for procurement, regulatory issues.

### Target group—severe malaria or severe anaemia

Defining the PDMC target group emerged as a key challenge and a rich discussion followed as participants sought to balance evidence against implementation feasibility. The evidence base and WHO recommendations for PDMC focus on children under 5 years of age hospitalized with severe anaemia. However, the current WHO chemoprevention recommendation leaves room for countries to tailor interventions to meet local needs, so it will be up to national decision-makers to determine the appropriate PDMC age range and indications, considering factors such as epidemiological data and implementation feasibility. For instance, Uganda has opted to target children under 5 years hospitalized with severe malaria even if they do not have concomitant severe anaemia (e.g. cerebral malaria), based partly on an earlier study conducted in Uganda [11] and on the operational feasibility of identifying severe malaria rather than severe anaemia. Conversely, in Malawi, it was noted that children with severe anaemia were readily identified due to severe illness and referred to district hospitals, where their haemoglobin would be tested and a blood transfusion administered. This highlights the importance of local contexts in defining and designing PDMC strategies. Given these shared experiences, there was agreement from all the countries that children with severe malaria without severe anaemia, such as those with cerebral malaria, should not be excluded from PDMC. The consensus was that the target group should be expanded to include children hospitalized with “severe anaemia *or* severe malaria”. Further expansion of the target group to include to school-age children due to the high burden of severe malaria was raised during the meeting, but further exploration by country is needed.

### PDMC delivery systems

Unlike other malaria prevention strategies there is no existing platform from which to deliver PDMC. It will be up to individual countries to design and implement a system, based on the drug choice, that fits within their local context. During the plenary sessions and in breakout groups the different delivery models for PDMC were discussed among the stakeholders, focusing on potential facilitators and barriers to implementation and adherence. Key considerations for adherence focused on both completion of the multiday drug regimen for each course and the monthly courses. Countries are at various stages in their thinking and planning around optimal delivery strategies for PDMC. In Uganda, the delivery strategy has already been selected, however, delegates indicated it could be refined based on lessons learned through implementation. In Malawi, researchers are conducting further implementation trials to optimize the preferred delivery model. Both Kenya and Benin are in the early phases of exploring delivery models and considering barriers and facilitators.

Presented below are the delivery strategies by country. Table 3 summarizes the pros and cons by strategy as discussed by participants. In addition, a full description of the delivery strategies presented by country is provided in Appendix 2.

**Table 3:** Key considerations for PDMC by delivery system

	Facility-based	Community-based	Self-initiated
	Caregiver collects PDMC drugs from a health facility	PDMC drugs are delivered to the caregiver at home or collected at a nearby location (e.g., local dispensary)	All PDMC drugs are given to the caregiver on discharge
PROS	Creates an opportunity for providers to check the child/touch base with caregiver on recovery Helps provider to monitor adherence to the monthly courses Monitoring for side effects and adverse events to drug	Reduced burden (financial) on caregiver because drugs are provided closer/delivered Could improve adherence as reduces access barriers Caregiver still has contact with health system via CHW/local dispensary	Reduces time and financial burden on caregivers Potentially improves adherence because the caregiver has all drugs Recent acceptability trial in Malawi indicated this was the preference of caregivers Potentially most cost-effective, feasible delivery model
CONS	Time and financial burden on caregiver to travel to the facility and collect drugs Failure of caregiver to return for subsequent courses due to burden—adherence issues	Additional workload for CHWs Relies on timely drug delivery to caregivers by CHWs Relies on strong linkages between discharging facility and CHWs Training requirements for CHWs	Fewer opportunities for providers to monitor adherence Caregiver-related issues (e.g., forgetfulness, sharing drugs with others, drugs lost)

Additional points raised:

Facility distances vary by country (i.e., what is considered ‘local’ and within communities)

Need to consider distances from health centres vs tertiary facilities (where children might have received treatment for severe anaemia)

How strong are the referral systems and links between larger facilities and local health centres and with CHWs

What mechanisms will be used to provide reminders (e.g., phone calls/SMS, home visits by CHWs)—considering feasibility, mobile phone ownership/use, existing structures utilised by other programmes (e.g., TB, HIV)

### Uganda (Speaker: Dr Gerald Rukundo, NMCP)

Uganda had already chosen to implement PDMC using a facility-based delivery model that would see the caregiver return monthly to the health facility to collect DP course-1, course-2, course-3. When possible, dose-1 of the monthly courses should be administered by directly observed therapy (DOT). Caregivers will receive monthly reminders to collect the PDMC drugs from a member of the VHT, who will be provided with a list of children from the health facility.

### Malawi (Speaker: Dr Lumbani Munthali, NMCP)

Malawi is further exploring community-based delivery of PDMC where caregivers are provided with course-1 at discharge from the facility and, subsequently, caregivers collect course-2 and -3 from their nearest health centre/village clinic each month. Ideally the child is also present and dose-1 can be administered by DOT. Dates for return visits will be written in the ‘Health Passport’ to serve as a reminder. However, additional research may be needed to evaluate the effect of other reminder strategies (e.g., SMS, phone calls) on adherence.

### Kenya (Speaker: Regina Kandie, NMCP)

Kenyan delegates were asked to consider what delivery mechanisms they would be interested in testing in the upcoming implementation trial, and they presented potential options that included facility, community, and self-initiated strategies. The facility-based strategy involved the caregiver returning to the discharge facility after 2 weeks to collect either all PDMC courses (for 3 months) or to collect course-1 only and then return monthly for course-2 and course-3. Two different community-based strategies were considered and involved monthly PDMC courses either to be collected from a local dispensary by the caregiver or delivered to them at home by community health promoters (CHPs). Caregiver reminders to collect from the local dispensary would be given by CHPs with links between the facility and local dispensary provided by the CHPs for the home-delivery model. A final self-initiated strategy was presented which would include all PDMC drugs (3 × monthly courses) provided to the caregiver on discharge, with monthly reminders to initiate the course by phone or CHP visit.

### Benin (Speaker: Manfred Accrombessi, IRCB)

Similarly, the delegates from Benin were asked to consider various delivery strategies. They presented options that included two self-initiated and one facility-based strategy. The self-initiated options both involved the caregiver being provided with all PDMC drugs (3 × monthly courses) alongside robust information and education communication (IEC) by healthcare providers, with the first option including monthly reminders to initiate the courses via either SMS, phone call or community health worker (*relais)* home visits and the second option having no reminders. The facility-based strategy consisted of the caregiver being provided with course-1 on discharge alongside IEC from healthcare providers and then asked to collect the remaining monthly courses from either a) the referral health facility or b) the sub-district health facility. No reminders would be provided to the caregivers.

### PDMC Indicators for Health Management Information Systems (HMIS)

Participants acknowledged the importance of capturing PDMC data and shared ideas on what indicators should be collected and how to incorporate them into existing data collection systems. The current thinking in Malawi is to track the number of PDMC courses completed through Health Passports, which are widely utilized. In addition, they aim to track coverage outcomes and impact indicators including adherence, reductions in hospital admissions, and number of deaths averted. It was also noted that HMIS needs to be tracking severe anaemia cases and data on blood transfusions. In Kenya, the electronic community health information system (eCHIS) is being rolled out and consideration is needed on how this will support any community-based delivery mechanism. From the breakout sessions, participants shared the key PDMC indicators that should be captured by routine data collection practices:

1. Number of children with severe anaemia.
2. Number of children with severe malaria.
3. Number of children who receive PDMC (coverage).
  - First course
  - Second course
  - Third course
4. Number of deaths averted.
5. Number of re-admissions reduced.
6. Trace blood transfusion data.

### Cost-effectiveness considerations

Determining the cost effectiveness of PDMC was perceived as an important piece of evidence required by decision-makers. Several indicators were mentioned by participants as being useful to help guide decision-making, including:

1. Cost savings related to PDMC.
2. Cost of implementation (e.g., training healthcare providers and CHWs, revision of tools, capacity building).
3. Cost per life saved.
4. Calculating real-time economic gains for PDMC.

### PDMC Market report (Speaker: Celine Audibert, MMV)

MMV undertook a market research study on perceptions of PDMC among stakeholders from five sub-Saharan African countries—Malawi, Kenya, Uganda, Nigeria, and Senegal. The findings highlighted existing knowledge gaps around the target group for PDMC (severe malaria rather than severe anaemia), the benefits and challenges with community versus facility delivery and concerns about drug resistance. In addition, the report summarizes the key potential barriers across the different ecological levels and highlights priority areas that need to be addressed to ensure the successful implementation of PDMC. At the national level, lack of political will and funding were perceived as important barriers to policy adoption and sustainability of PDMC. Significant health system constraints that may hamper the implementation of PDMC include the availability of PDMC drugs and, supply chain and logistical shortcomings. Study participants indicated that a stable supply of drugs and effective distribution channels would be essential, and that data collection and management systems would need to be robust. At the facility and community level, potential barriers included a lack of awareness about PDMC, inadequately trained healthcare providers, poorly motivated and resourced CHWs and ineffective facility to community linkages for follow-up mechanisms. Potential implementation hurdles at the end user level, such as poor adherence to PDMC courses/doses, caregiver hesitancy related to limited awareness about PDMC and lack of support from community influencers could be significant barriers that would require mitigation strategies.

## Conclusions and outcomes

### Next steps for countries

The policy roadmap for each country reflects the current planning stage and additional evidence requirements by decision-makers to move forwards. Country representatives outlined the next steps which involved a range of activities including stakeholder engagement, ongoing evidence generation, internal policy processes, and implementation activities, presented below in Table 4.

**Table 4:** Next steps on the policy adoption pathway by country

Uganda	PDMC is included in the malaria treatment policy and Integrated Management of Malaria training tools PDMC has been integrated into Global Fund grant application—which has been approved PDMC drug requirements have been quantified Roll out will include VHT training and provision of job aids Sensitization of VHTs and the wider community
Malawi	Conducting PDMC implementation studies to optimise delivery strategy Present PDMC results at the next Health Services Delivery TWG for policy adoption Present TWG recommendations to MoH senior management for new policy approval Phased roll out of PDMCC in 10 selected districts with scale up over coming years
Kenya	Presentation on PDMC to key stakeholders through the Committee of Experts on case management TWG Formation of sub-group (within the TWG) specifically for PDMC Review of national policy guidelines
Benin	Awaiting outputs from formative research Conduct national stakeholder meetings and co-design workshop Evidence from trials—both effectiveness of intervention and delivery system feasibility Local evidence is required to persuade policy makers that PDMC is effective and feasible

### Research agenda

Current and upcoming PDMC research was discussed among stakeholders during the meeting including studies being conducted as part of the PDMC Saves Lives project (formative research in all four countries and implementation trials in Benin and Kenya), other relevant research (PDMC-implement trial in Malawi) and the identification of additional research needs to be considered.

In all four countries:

1. Formative research exploring perceptions around key components of PDMC:
  - Drug choice and alternatives
  - Delivery strategies (e.g., facility, community, self-initiated)
  - Barriers and facilitators to adherence
  - Barriers and facilitators to implementation.

In Benin, Kenya (funded by Global Health EDCTP3 Joint Undertaking), and Malawi (funded by Norwegian Research Council, working in partnership with EDCTP):

2. Implementation trials based on country-specific delivery strategies with outputs including:
  - Acceptability of delivery strategies
  - Feasibility of delivery strategies
  - Adherence to PDMC courses/doses
  - Cost-effectiveness.

Additional research items identified:

3. Clinical study to determine appropriate PDMC target group (i.e., age range/underlying condition)—including cost effectiveness.
4. Uganda/Malawi—study to explore optimal coordination mechanisms between health facilities and community structures for follow-up.

## Data Availability

No datasets were generated or analysed during the current study.

## Appendix

Appendix 1. Questions provided to country breakout session groups.

**Table:**

Question 1	Which drugs are/will be used for PDMC? Why? If more than one drug is being considered, list and rank drug(s) of choice Considerations: • Consider treatment context—first line treatment including any multiple first-line treatment (MFT) strategies; drug resistance; other relevant factors • Consider prevention context—drugs used for SMC, PMC, and other chemoprevention • Choice of drug will determine PDMC schedule start e.g., 2 weeks if AL and 4 weeks if DP
Question 2	Which delivery strategies or platforms will or may be used for delivering PDMC? Why? Identify at least two preferred alternative strategies to evaluate in a trial (Benin and Kenya) Considerations: • Consider delivery of the drug (self-administered by caregiver, CHW-initiated or hybrid) • Consider coordination between caregiver, community and facility/health system • Identify potential challenges and mitigations for each
Question 3	What economic evidence do we need to generate to convince the policy and financial decision-makers to implement PDMC at scale?
Question 4	What are the next steps for country x regarding PDMC?

Appendix 2: PDMC delivery strategies discussed in the breakout sessions by country.

**Table:**

	Facility	Community	Self-initiated
Uganda	• DP given at discharge • Caregiver returns to the facility to collect monthly courses of DP • Dose-1 ideally taken by DOT • Dose-2 and dose-3 self-administered at home • Follow-up conducted by VHTs who will be provided with a list of children from the health facility		
Malawi		• DP given at discharge • Caregiver returns to local community/village clinic each month to collect courses of DP (course-1, course-2, course-3) • Dose-1 is taken by DOT, with dose-2,3 self-administered at home • Date in Health Passport to serve as a reminder with other models being tested (e.g. SMS, phone calls, home visits)	
Kenya	• AL given at discharge • Caregiver returns to the tertiary facility (where they were discharged) 2 weeks after discharge and receives either: • All PDMC courses (for 3 months) • Course-1 only and they return to the facility each month to collect course-2, course-3	Option 1: • AL given at discharge • Caregiver returns 2 weeks after discharge to collect course-1 of PDMC at local dispensary • Caregiver returns monthly to collect other courses • Dose-1 ideally taken by DOT • Reminders by CHP (community health promoter) and CHP providers of the drugs with CHA provided linkage Option 2: • AL given at discharge • Monthly PDMC courses are delivered each month to the caregiver at home by a CHV • CHVs receive monthly reminders to collect/deliver drugs from the local dispensary	• AL given at discharge • All PMDC courses given to caregivers at discharge from the facility • Monthly reminders by phone/CHV
Benin	• Course-1 PDMC drugs are given to the caregiver alongside IEC from the HCW • Caregiver instructed to return monthly and collect course-2, course-3 from either: • Referral health facility • Sub-district health facility		Option 1 • All drugs given to caregiver at discharge • IEC prior to discharge • Monthly courses are self-initiated • Reminders via CHW/SMS/phone call Option 2 • All drugs given to caregiver at discharge • IEC prior to discharge • Monthly courses are self-initiated • No reminders are provided

## Abbreviations

AL: Artemether-lumefantrine
ASAQ: Artesunate-amodiaquine
CHP: Community health promoter
CHV: Community health volunteer
CHW: Community health worker
DP: Dihydroartemisinin-piperaquine
HMIS: Health Management Information Systems
HSA: Health surveillance assistant
IPTp: Intermittent preventive treatment in pregnancy
IRCB: Institut de Recherche Clinique du Benin
IRD: Institut de recherche pour le development
KEMRI: Kenya Medical Research Institute
CDC: US Centers for Disease Control
LSTM: Liverpool School of Tropical Medicine
NMCPs: National Malaria Control Programmes
MMV: Medicines for Malaria Venture
MoH: Ministry of Health
PMC: Perennial malaria chemoprevention
PDMC: Post-discharge malaria chemoprevention
PMI: President’s Malaria Initiative
SMC: Seasonal malaria chemoprevention
SP: Sulfadoxine-pyrimethamine
SMS: Short message service
SPAQ: Sulfadoxine-pyrimethamine with amodiaquine
TRUE: Training and Research Unit of Excellence
TWG: Technical Working Group
VHT: Village Health Team
WHO: World Health Organization
